# Exploring friendship quality and the practice of savoring in relation to the wellbeing of Greek adults

**DOI:** 10.3389/fpsyg.2023.1253352

**Published:** 2023-10-06

**Authors:** Christos Pezirkianidis, Maria Christopoulou, Evangelia Galanaki, Kalliope Kounenou, Eirini Karakasidou, Dimitra Lekka, Antonios Kalamatianos, Anastassios Stalikas

**Affiliations:** ^1^Lab of Positive Psychology, Department of Psychology, Panteion University of Social and Political Sciences, Athens, Greece; ^2^Lab of Psychology, Department of Primary Education, National and Kapodistrian University of Athens, Athens, Greece; ^3^Department of Education, School of Pedagogical and Technological Education, Marousi, Greece; ^4^Department of Education, School of Education, University of Nicosia, Nicosia, Cyprus

**Keywords:** adult friendship, friendship quality, savoring, wellbeing, adults

## Abstract

Previous research findings demonstrate that both savoring ability and the presence of high-quality friendships play a significant role in enhancing one’s overall sense of wellbeing. However, these associations have not been thoroughly investigated within a diverse range of adults across their lifespans, nor have they been explored in the specific cultural context of Greece. Thus, the primary objective of this study was to delve into the relationships between close friendship quality, the utilization of savoring techniques, and wellbeing within the Greek cultural framework. The study involved 771 adults from Greece with an average age of 38.35 years, who completed the McGill Friendship Functions Questionnaire, the PERMA Profiler, and the Abridged Ways of Savoring Checklist. Results revealed that there exists a positive correlation between friendship quality and savoring strategies with overall wellbeing. Moreover, the study identified a significant association wherein a greater employment of savoring strategies was linked to higher levels of friendship quality. While this study contributes valuable insights, it also has limitations that warrant acknowledgment. Furthermore, suggestions for potential future research directions are proposed, and the implications of these findings are discussed in relation to interventions aimed at enhancing both friendships and the practice of savoring across various contexts.

## Introduction

### Adult friendship and wellbeing

Friendship is a voluntary, reciprocal, informal, and unconstrained close relationship between two unique peers that endures over time, facilitates the socioemotional goals of both parties, yields shared benefits, and is typically characterized by intimacy, emotional warmth, support, and trust (Wrzus et al., 2017; Farmer and Kali, 2018). Individuals seek a variety of features and specific provisions in their friendships that describe their friendship quality (Demir and Weitekamp, 2007). According to Mendelson and Aboud (1999), the following six functions are fulfilled in high-quality friendships: stimulating companionship, help, intimacy, reliable alliance, emotional security, and self-validation. *Stimulating companionship* involves doing enjoyable things together, while *emotional security* includes comfort and reassurance provision in stressful situations that cause fear, anxiety, or anger. Also, *reliable alliance* refers to knowing that one can count on one’s friend, and *help* refers to support, assistance, and guidance provision. Finally, *self-validation* is defined as encouragement to maintain a positive self-image, and *intimacy* refers to self-disclosure (Fehr and Harasymchuk, 2018).

Friends are among the most significant interpersonal bonds in life. They largely fulfill the fundamental human need for belonging and social interaction (Hojjat et al., 2017). The development of a friendship is valuable throughout adulthood, as it significantly contributes to individuals’ positive adaptation to new situations and adversity (Buote et al., 2007). A recent systematic review indicated that several adult friendship variables are related to wellbeing and its components, especially friendship quality, number of friends, attempts to maintain friendship, socializing with friends, and support from friends (Pezirkianidis et al., 2023). Among these variables, friendship quality has been found to facilitate increased positive emotions, self-worth, happiness, social competence, accomplishments, meaning in life, and psychological adjustment (e.g., Demir and Weitekamp, 2007; Koestner et al., 2012; Akin and Akin, 2015; Carmichael et al., 2015; Secor et al., 2017). However, most studies that focus on the associations between friendship quality and wellbeing indices are based on emerging adult and university student samples (Pezirkianidis et al., 2023).

Wellbeing is based on five pillars according to Seligman’s (2011) multidimensional conceptualization. The experience of positive emotions represents the emotional nature of wellbeing and broadens the repertoire of actions and thoughts (Fredrickson, 2004). Engagement refers to increased interest and absorption in everyday tasks and activities (Kern et al., 2015). Positive relationships represent the social facet of wellbeing; high-quality and satisfying relationships lead to perceived support from others, which predicts greater physical and psychological wellbeing (Chandoevwit and Thampanishvong, 2016; Mitskidou et al., 2021). Meaning reflects the belief that life is worth living and includes feelings of connection to a greater entity (Kern et al., 2015). Finally, accomplishment refers to the achievement of ordinary goals and boosts individuals’ life satisfaction and psychological wellbeing (Nohria et al., 2008).

### Savoring, interpersonal relationships, and wellbeing

Savoring is the tendency to focus on and enjoy past, present, and future positive experiences so that the pleasure they bring is intensified (Bryant and Veroff, 2007). There are behavioral savoring strategies, such as physical manifestation of positive emotions and sharing positive experiences with others, and cognitive savoring strategies, for example, congratulating oneself, getting fully absorbed in the moment, filling intentionally one’s memory with diverse aspects of a positive event or counting blessings (Bryant and Veroff, 2007; Quoidbach et al., 2010).

Research suggests that individuals who use savoring techniques report greater wellbeing, more frequent experiences of positive emotions, stronger connections with others, deeper engagement in activities, and enhanced purpose in life (Gable et al., 2004; King et al., 2006; Bryant and Veroff, 2007). Savoring facilitates the experiencing of positive emotions and thus acts as a driving force for all the beneficial effects of positivity (Bryant and Veroff, 2007; Kiken et al., 2017). Based on the Broaden-and-Build Theory, experiencing positive emotions undoes the unpleasant effects of negative emotions, broadens the repertoire of thoughts and actions, and builds time-resistant resources, which in turn enhance life satisfaction and longevity, increase the possibility of experiencing future positive emotions and strengthen resilience to adversity (Fredrickson, 2004; Tugade and Fredrickson, 2004; Fredrickson and Branigan, 2005). In addition, research findings show that high-savoring individuals experience more positive events in their daily lives or perceive them as such (Hurley and Kwon, 2013).

Savoring also applies to interpersonal relationships. Savoring close relationships enhances the sense of unity and belonging by creating a special bond between its members, which itself is an object of savoring (Bryant and Veroff, 2007). Additionally, increased trait savoring has been found to facilitate willingness to form new relationships, such as friendships (Harrison, 2014). To add more, recent studies focus on the effects of relational savoring, i.e., the process of savoring past, present, or future experiences or moments of connectedness with significant others (Froidevaux et al., 2023), on wellbeing indices. Experimental studies on relational savoring have shown its effects on both relational and personal wellbeing indices. For example, partners in long-distance relationships who savor positive relational experiences feel more positive and less negative emotions (Borelli et al., 2015), while parents of infants and toddlers who participated in a relational savoring intervention felt more satisfied with the relationship with their children (Burkhart et al., 2015). Moreover, relational savoring has been found to moderate the relationship between psychological distress and relationship satisfaction of military partners who are non-deployed (Froidevaux et al., 2023). Additionally, it has been found to reduce the negative affect and attachment anxiety of youth in residential treatment (Wang et al., 2021). Taking everything into account, savoring in interpersonal relationships facilitates greater attachment security, more capacity to understand the mental states of the self and others, increases in positivity and meaning derived from relationships, and greater relationship quality and satisfaction (Borelli et al., 2020). The aforementioned studies highlight the contribution of savoring in parent–child and romantic relationships but there are no findings regarding the association between savoring and positive experiences in friendships.

### The present study

The present study aims to investigate the relationship between adult friendship quality indicators, savoring, and wellbeing using a lifespan Greek adult sample. This study is considered to add to the existing literature, since the association between adult friendship quality, savoring, and wellbeing has never been examined in the Greek cultural context, while the relationship between savoring and friendship quality remains understudied. Moreover, the relationship between adult friendship quality and wellbeing has been mainly examined in young adults and not in a lifespan adult sample.

The research hypotheses are the following:

1. High friendship quality, as expressed by the fulfillment of the six functions, namely stimulating companionship, help, intimacy, reliable alliance, emotional security, and self-validation, will be positively correlated with wellbeing.
2. Savoring will be positively correlated with friendship quality.
3. Savoring will be positively correlated with wellbeing.

## Methods

### Participants

The sample consisted of 771 Greek adults (*M*_age_ = 38.35 years, *SD* = 13.33, age range 18–73). Of them, 58.9% were women, 47.1% were married, 64.2% lived in Attica, Greece, and 71.6% were employed, while 32.5% were high school graduates and 45.6% were university graduates (see Table 1).

**Table 1:** Participant’s sociodemographic characteristics (*N* = 771).

Variable	*N*	*%*
Gender		
Male	317	41.1
Female	454	58.9
Marital status		
Single/unmarried	341	44.2
Married	363	47.1
Divorced	54	7.0
Widowed	13	1.7
Work status		
Employed	552	71.6
Unemployed	214	27.8
Residence		
Attica	495	64.2
Other Greek regions	269	34.9
Missing	7	0.9
Educational level		
Secondary education	250	32.5
University students	70	9.1
University graduates	351	45.5
Master’s degree	91	11.8
PhD	8	1.0
Missing	1	0.1

### Measures

#### Friendship quality

The McGill friendship questionnaire – friendship functions (MFQ-FF; Mendelson and Aboud, 1999) consists of 30 items scored on a 9-point Likert-type scale ranging from 0 (*never*) to 8 (*always*) and assessing the degree to which the individual’s close friendship fulfills the six friendship functions: stimulating companionship, help, intimacy, reliable alliance, emotional security, and self-validation. Sample items are the following: “___ helps me when I need it.,” “___ makes me laugh.,” and “___ compliments me when I do something well.” (___ is filled with a close friend’s name). Scale means were computed to create an overall close friendship quality score. The psychometric properties of the scale have been examined in other cultural contexts (e.g., Souza and Hutz, 2007; Wagner, 2019) including Greece (Pezirkianidis, 2020; Pezirkianidis et al., 2022) indicating adequate construct validity and internal consistency.

#### Wellbeing

The PERMA profiler (Butler and Kern, 2016) consists of 23 items (three items per component, and extra single items, e.g., loneliness) measuring the following wellbeing components: positive emotions, engagement, positive relationships, meaning, and accomplishment. Sample items are the following: “In general, to what extent do you lead a purposeful and meaningful life?,” “In general, how often do you feel joyful?,” and “Taking all things together, how happy would you say you are?.” The scale is scored on an 11-point Likert-type scale ranging from 0 (*never/not at all/terrible*) to 10 (*always/completely/excellent*). An overall wellbeing score is computed and was used in the present study. The psychometric properties of the scale have been examined in several cultural contexts (e.g., Wammerl et al., 2019; Bartholomaeus et al., 2020) including Greece (Pezirkianidis et al., 2021) indicating adequate construct validity, internal consistency, and test–retest reliability.

#### Savoring

The *Abridged Ways of Savoring Checklist for Adults* (WOSC; Chadwick, 2012; Greek version: Pezirkianidis, 2020) is Bryant and Veroff’s (2007) scale short form and consists of ten positive and negative savoring strategies scored on a 7-point Likert-type scale ranging from 1 (*totally disagree*) to 7 (*totally agree*). Sample items are the following: “I looked for other people to share it with.,” “I told myself how proud I was.,” and “I reminded myself how lucky I was to have this good thing happen to me…” In the present study, only the total score on positive savoring strategies was used. The psychometric properties of the scale have been examined in several cultural contexts (e.g., Kim and Bryant, 2017; Miyakawa et al., 2019) including Greece (Pezirkianidis, 2020) indicating adequate construct validity and internal consistency reliability.

#### Demographics

Participants reported their gender, age, marital status, residence, educational level, employment status, and number of close friends.

### Procedure

Data was collected during October–December 2019. Students from Panteion University of Social and Political Sciences, Athens, Greece recruited, after receiving training, adults of their social milieu. Before providing their consent, participants were informed about the aims of the study and the anonymity of their responses. No external incentives or compensation were provided to them. The data was recorded on answer sheets, scanned using the Remark Office OMR (Gaikwad, 2015), and analyzed using the IBM Statistical Package for Social Sciences 21 (Hinton et al., 2014). Cases with missing data in the main measures were excluded (*N* = 35) after confirming randomness, i.e., participants that answered the highest, lowest, or the same value in all items.

## Results

Shapiro–Wilk tests showed that all variables were skewed. Descriptive statistics (see Table 2) and reliability analysis were performed, with all subscales demonstrating adequate internal consistency (Cronbach’s α = 0.86–0.97 > 0.70; Galanakis et al., 2017).

**Table 2:** Spearman’s Rho correlation coefficients and descriptive statistics for study variables (*N* = 771).

Variables	1	2	3	4	5	6	7	8	9	Mean	SD
1. Stimulating companionship	1									31.23	7.31
2. Help	0.78	1								30.54	6.99
3. Intimacy	0.73	0.78	1							33.04	6.68
4. Reliable alliance	0.67	0.69	0.82	1						34.39	6.30
5. Emotional security	0.76	0.84	0.86	0.76	1					31.54	6.86
6. Self-validation	0.75	0.75	0.75	0.68	0.79	1				30.94	6.85
7. Friendship quality	0.87	0.90	0.90	0.82	0.93	0.86	1			6.35	1.18
8. Savoring	0.46	0.43	0.40	0.37	0.40	0.45	0.47	1		60.18	12.39
9. Overall wellbeing	0.50	0.50	0.44	0.43	0.44	0.50	0.48	0.55	1	6.91	1.43

All correlations were significant at the 0.001 level.

Most participants reported a same-sex friend (females: 90.7%, males: 88.6%), and 58.1% said that their closest friend was a female. Also, participants reported a mean number of three close friends. Spearman’s *Rho* correlations (see Table 2) showed that wellbeing had statistically significant positive correlations with all friendship functions and savoring, which also correlated positively with all friendship functions. The friendship functions that were most associated with wellbeing and savoring were stimulating companionship, help, and self-validation.

Two stepwise multiple regression analysis were conducted to explain the variance of wellbeing and friendship quality (see Table 3). The results indicated that gender and age (step 1) explain only 1.7% of wellbeing variance [*F*(2, 769) = 8.349, *p* < 0.001], indicating greater wellbeing among younger participants. At step 2, the effects of age and gender became insignificant, while higher stimulating companionship, help, reliable alliance, and self-validation in close friendship were associated with greater wellbeing [*F*(8, 763) = 47.050, *p* < 0.001, *R^2^* = 0.29]. At step 3, findings indicate that 40% of the wellbeing variance can be significantly and positively predicted by higher friendship quality (help, reliable alliance, and self-validation), greater savoring strategies, and male gender [*F*(9, 762) = 70.291, *p* < 0.001]. Moreover, the findings show that 23% of friendship quality variance can be accounted for by the use of savoring strategies [*F*(1, 770) = 288.489, *p* < 0.001].

**Table 3:** Multiple regression analyses for the prediction of wellbeing and friendship quality (*N* = 771)

Predictors	*b*	*SE b*	Β	*p*
(Model 1: Wellbeing)	7.11	0.22		<0.001
Step 1: Age	−0.01	<0.01	−0.11	<0.001
Gender	0.16	0.09	0.05	0.096
Step 2: Age	0.00	<0.01	−0.03	0.232
Gender	−0.11	0.08	−0.04	0.182
Stimulating companionship	0.03	0.01	0.17	<0.001
Help	0.04	0.01	0.17	0.002
Intimacy	0.00	0.01	−0.03	0.652
Reliable alliance	0.03	0.01	0.14	0.005
Emotional security	−0.02	0.01	−0.10	0.107
Self-validation	0.05	0.01	0.24	<0.001
Step 3: Age	0.00	<0.01	−0.01	0.624
Gender	−0.27	0.08	−0.09	0.001
Stimulating companionship	0.02	0.01	0.08	0.090
Help	0.03	0.01	0.13	0.012
Intimacy	−0.01	0.01	−0.03	0.570
Reliable alliance	0.03	0.01	0.11	0.011
Emotional security	−0.02	0.01	−0.07	0.213
Self-validation	0.04	0.01	0.18	<0.001
Savoring	0.05	<0.01	0.40	<0.001
(Model 2: Friendship quality)	3.55	0.17		<0.001
Savoring	0.05	<0.01	0.48	<0.001

## Discussion

The present study aimed to shed light on the relationship between friendship quality, savoring, and wellbeing among Greek adults. Findings showed that all friendship functions were positively correlated with wellbeing. This finding is in accordance with existing data supporting the positive associations between friendship quality and wellbeing (e.g., Demir et al., 2017; Holt-Lunstad, 2017; Pezirkianidis et al., 2023). As expected, stimulating companionship, self-validation (e.g., Demir and Weitekamp, 2007), and help (e.g., Secor et al., 2017) were the friendship functions that were significantly associated with increased wellbeing. Previous studies have also shown positive links between these functions, increased life satisfaction (Chandoevwit and Thampanishvong, 2016), and positive emotions (Kok et al., 2013). Taking everything into account, spending time with a close friend, doing enjoyable things together, receiving emotional and instrumental support and encouragement to maintain a positive self-image relate to greater wellbeing among Greek adults, thus, the first hypothesis of this study has been confirmed (Demir, 2015).

Moreover, the second research hypothesis has been confirmed, since the use of savoring strategies is positively associated with greater friendship quality and specific friendship functions, especially stimulating companionship, help, and self-validation. This is a new finding in relevant literature since previous studies have focused on the relationships between savoring and other close interpersonal bonds, such as parent–child and romantic relationships (Borelli et al., 2020). When savoring is applied in close relationships enhances the sense of unity and belonging by creating a special bond between the two members, increases the relational positivity and meaning, deescalates the relational distress, and facilitates greater attachment security, relationship quality, and satisfaction (Bryant and Veroff, 2007; Borelli et al., 2020; Wang et al., 2021; Froidevaux et al., 2023).

The third hypothesis of the present study was also confirmed since savoring was found to have a strong connection with wellbeing of Greek adults. This is in line with previous findings indicating that the use of savoring strategies facilitates the experiencing of positive emotions (Bryant and Veroff, 2007). This, in turn, broadens the repertoire of thoughts and actions and builds enduring resources that enhance meaning in life, life satisfaction and facilitate wellbeing (Fredrickson, 2000, 2004; Tugade and Fredrickson, 2004; Fredrickson and Branigan, 2005). Moreover, the findings of the regression analysis indicate that savoring accounts for variance in wellbeing over and above friendship indices, since savoring enhances social wellbeing by increasing the sense of belonging, positive relational experiences, and relational resilience (Borelli et al., 2020; Froidevaux et al., 2023). Close interpersonal bonds with such characteristics are a main source of meaning in life (Russo-Netzer, 2019). Taking everything into consideration, there are many paths through which savoring becomes beneficial to individuals’ wellbeing.

Finally, the findings provided insight into the associations between gender, age, and wellbeing. In contrast to previous findings (e.g., Bruine de Bruin et al., 2020), we found that younger age is associated with greater wellbeing in the Greek context. In addition, age effects became insignificant when friendship indices were added into the equation, which is in line with previous findings highlighting that social satisfaction, i.e., the perception of relationship quality, becomes more important than age when explaining wellbeing during adulthood (Bruine de Bruin et al., 2020). On the other hand, gender was not significantly associated to overall wellbeing levels in the Greek context. This finding provides fruitful information since previous findings regarding the gender differences in overall wellbeing are inconsistent (Matud et al., 2019). However, when both friendship indices and savoring were added into the equation, gender effects became significant indicating that the male gender was associated with greater wellbeing. This finding is not consistent with previous ones, since current literature indicates that women use more savoring strategies compared to men, such as sharing positive events with others, expressing positive feelings through words and actions, and counting blessings. Men, on the other hand, tend to use more dampening techniques (Bryant and Veroff, 2007). Also, men seem to score higher than women in self-acceptance and autonomy, and women score higher in positive relations with others (Matud et al., 2019). Future studies need to shed more light on this relationship.

### Limitations and suggestions for future research

The present study was a single timepoint, self-report survey that assessed only constructs of positive valence based on Likert-type scales. Thus, participants’ responses could have been due to several response biases, e.g., social desirability, positive response bias, or loyalty to friends. Furthermore, the snowball sampling technique resulted in recruiting individuals from a restricted network of individuals. Moreover, several sociodemographic variables, such as marital status, educational level, or parenthood, were not considered. Additionally, data were only collected from one member of the friendship, therefore we only have a one-sided viewpoint of the relationship. Finally, possible differences between same- or cross-gender friendships were not examined in this study.

Future studies could focus on the specific dimensions of wellbeing or draw on different multidimensional conceptualizations of wellbeing, e.g., Keyes (2005) model, to explain its associations with positive friendships and savoring. Furthermore, qualitative or mixed-method studies, data collection from both friendship members, and longitudinal research designs are needed for an in-depth exploration of this issue, especially in the Greek context, where close relationships with both friends and family members are reported. Recently, the COVID-19 pandemic and three consecutive long-term lockdowns have largely impacted human relationships in Greece and have created the need for further research on the role of friendships and savoring in wellbeing and on the mechanisms underlying these associations. Also, the design, implementation, and evaluation of new interventions focusing on positive friendship development across adulthood is an important aim of future research. Finally, the present study could be replicated in other cultural contexts; cross-cultural comparisons would shed light on how positive friendship experiences and savoring practices explain wellbeing levels in different contexts.

### Contributions of the findings

The findings of the present study uniquely contribute to the understanding of the relationships between close friendship quality and wellbeing during adulthood and not exclusively among emerging adults. Additionally, this study is one of the first attempts to understand the relationships between friendship dimensions and savoring practices. These contributions become even more significant since this was the first study in Greece that underlines the importance of both savoring and friendship quality to Greek adult’s wellbeing, whose mental health seems to be strongly connected to their social satisfaction (Malikiosi-Loizos and Anderson, 1999).

Moreover, the findings of this study have important practical implications. Positive education practices to cultivate savoring and positive friendships could be incorporated in several settings. Mental health professionals, coaches, social workers, and educators in school, work, or clinical settings could design and apply interventions to enhance people’s skills in savoring positive experiences and building positive friendships. This is expected to increase people’s wellbeing, social competence, psychological resilience, sense of meaning in life, and positive emotions.

## Data availability statement

The raw data supporting the conclusions of this article will be made available by the authors, without undue reservation.

## Ethics statement

Ethical approval was not required for the studies involving humans because the Ethics Committee of the Panteion University had not been established when the research took place. The studies were conducted in accordance with the local legislation and institutional requirements. The participants provided their written informed consent to participate in this study.

## Author contributions

CP: Conceptualization, Data curation, Formal analysis, Investigation, Methodology, Project administration, Resources, Software, Supervision, Validation, Visualization, Writing – original draft, Writing – review & editing. MC: Visualization, Writing – original draft. EG: Visualization, Writing – review & editing. KK: Funding acquisition, Writing – review & editing. EK: Project administration, Writing – review & editing. DL: Project administration, Writing – review & editing. AK: Project administration, Writing – review & editing. AS: Conceptualization, Supervision, Validation, Writing – review & editing.
